# Worldwide research on fear of childbirth: A bibliometric analysis

**DOI:** 10.1371/journal.pone.0236567

**Published:** 2020-07-29

**Authors:** Lijing Dai, Na Zhang, Liu Rong, Yan-Qiong Ouyang

**Affiliations:** School of Health Sciences, Wuhan University, Wuhan, China; University of Sydney, AUSTRALIA

## Abstract

**Objective:**

To review the research on fear of childbirth, analyze and evaluate the publications by means of bibliometric analysis, and provide suggestions and scopes for future study.

**Data sources:**

Web of Science, PubMed, Embase and the Cochrane Library databases.

**Study selection:**

All published articles focusing on the fear of childbirth from inception to February 10, 2020.

**Data extraction:**

A total of 743 articles were included for final analysis. Bibliographic data were exported from databases and then cleaned manually before using Microsoft Excel and VOSviewer to analyze and visualize the findings.

**Data synthesis:**

It was found that 743 articles have been cited 31515 times (h-index: 98). The volume of publications increased by 29.3 times in the past two decades. Across the globe, Sweden was the most prolific country having 129 articles (17.4%) with six of its institutions in the top ten most prolific institutions in the world. Fifty-two (7.8%) documents were published in *Midwifery* journal. The most prolific author was Hildingsson, who published 35 articles (4.7%). “Cesarean section,” “experience,” and “factor” were the words that appeared most frequently in titles and abstracts of studies. “Point prevalence of psychiatric disorders during the second trimester of pregnancy: A population-based study” was the most cited article and received 525 citations.

**Conclusion:**

There is an increasing interest in the research in fear of childbirth during the past two decades. This study has demonstrated that the Swedish authors have a leading role on this topic. Researchers especially in countries with high birth rates, need to promote research projects in this field as it is an important public health issue.

## Introduction

It has been found through a study that women often face psychological distress and anxiety when confronted with the act of giving birth [1], which leads to a fear of childbirth (FOC) in them. FOC is a common psychological phenomenon before, during and after pregnancy which refers to a state of intense anxiety [1, 2]. The degree of FOC ranges from mild to severe; where a mild fear is insignificant while severe or extreme fear needs clinical intervention [3–5]. According to previous studies, FOC occurred in approximately 20% of pregnancies [6, 7]. Moreover, the worldwide prevalence of severe FOC has seen an upward trend from 14% in the recent years [8].

Although FOC is a common psychological problem, it ushers in a series of adverse consequences affecting women’s health, well-being and daily activities [9]. Insomnia, nightmares, fatigue and extreme insecurity may occur as FOC gets worse [10–12] causing some women to even opt for the termination of pregnancy [10, 11]. FOC not only increases the risk of dystocia and prolongs labor [13], but also adds to the likelihood of postpartum depression and post-traumatic stress disorder (PTSD) [14]. Furthermore, women having severe FOC may doubt their ability to cope with childbirth, resulting in a cesarean birth without medical indications [15, 16].

A clinical guideline on the management of antenatal and postnatal mental health from the National Institute for Health and Care Excellence (NICE) advocated that the mental health of pregnant women should be treated as importantly as physical health in prenatal and postnatal care [17]. Given the high prevalence of FOC and its negative consequences worldwide, it unequivocally deserves ample focus. However, FOC has received limited attention compared to other mental health problems during pregnancy, such as postpartum depression. Postpartum depression is routinely screened in clinics, whereas FOC and other anxiety-related disorders are neglected [18]. In addition, FOC is a complex psychological problem without consensus on its definition and measurement [3], on which limited attention has been paid, especially in countries like China [2]. Therefore, there is a need to provide an informed and expanded description of this issue in published literature.

Bibliometrics is an important quantitative analysis approach using mathematical/statistical methods to evaluate the quality and quantity of published papers and assess worldwide research productivity in a particular field [19]. Research output plays an important role in scientific development and provides a key link for the generation and utilization of knowledge [20]. In addition, bibliometric analyses estimate the impact of existing academic achievements in a scientific community, and investigate a general trend of a specific theme [21, 22]. Therefore, a bibliometric analysis was applied in this study to give an insight into the current state of FOC, evaluate its worldwide research productivity, development, and trends in research on FOC. Furthermore, this study aims to provide objective information and direction for planning research and development programs in this area. Hopefully, this will stimulate researchers in the field of obstetrics and gynecology in all countries to place added emphasis on FOC and mental health care for women.

## Methods

### Search strategy

We searched the electronic databases including Web of Science, PubMed, Embase and the Cochrane Library for the publications on FOC from the inception of each database to February 10, 2020. The search term was integrated as follows: “TI = (((“childbirth” OR “birth” OR “delivery” OR “labor” OR “labour”) AND (“fear”)) OR “tokophobia” OR “tokophobia”) OR AB = (((“childbirth” OR “birth” OR “delivery” OR “labor” OR “labour”) AND (“fear”)) OR “tokophobia” OR “tokophobia”) (See details in S1 Appendix). The publications were carefully reviewed by two independent reviewers to ensure that the entries were relevant to the topic. Disagreements were discussed and resolved by consensus with a third reviewer. The bibliographic information for irrelevant articles were removed from the dataset. In order to reduce repetition and to better explore the development of original research, publications such as meeting abstract, review, proceedings paper, editorial material, letter, correction, and news item were excluded.

### Data preprocessing

Due to different versions of spelling in this topic, one country/institution/author/journal might be categorized into multiple countries/institutions/authors/journals. Therefore, it is necessary to preprocess the data in order to achieve accurate results [23]. In the first step, the authors set a uniform export format in Endnotes (e.g. authors’ name format: Last name, A. B.; journal name format: use of full journal name). Next, data was exported to Microsoft Excel for manual data cleaning. The data were cleaned by two researchers, a process that included finding the missing value, merging different spellings of the same country/institution (e.g. Hong Kong, Macao, Taiwan all belong to China; Tartu Univ and Univ Tartu are the same institution). In order to avoid counting a journal article more than once due to the number of authors, the countries and institutions were analyzed following the first author's country and institution. The next step was word preprocessing. The terms were extracted using VOSviewer software (Leiden University, Leiden, Netherlands) from titles and abstracts. The search-related terms (e.g. fear of childbirth, childbirth fear) and general terms (e.g. pregnant women, study, sample) were omitted, and the synonymous keywords (e.g. caesarean section, cesarean section, caesarean birth) were merged into one word.

### Data analysis

Article citations were obtained using Google Scholar on February 19, 2020. The citation report, trends of publications and the prolific countries/institutions/authors/journals were analyzed using PivotTable in Microsoft Excel. Word frequency analyses were visualized by VOSviewer, which is a visualization software freely available at www.vosviewer.com [24]. The bibliometric network is based on distance and composed of circles and lines. The circles represent words in the current study, and a larger circle indicates the item appears more often in this field [24, 25]. A line indicates that a relationship exists between two circles (co-occurrence relation), and a smaller distance and thicker line indicates a stronger relationship [24–26].

## Results

### Description and trends of publications

**Fig 1:**
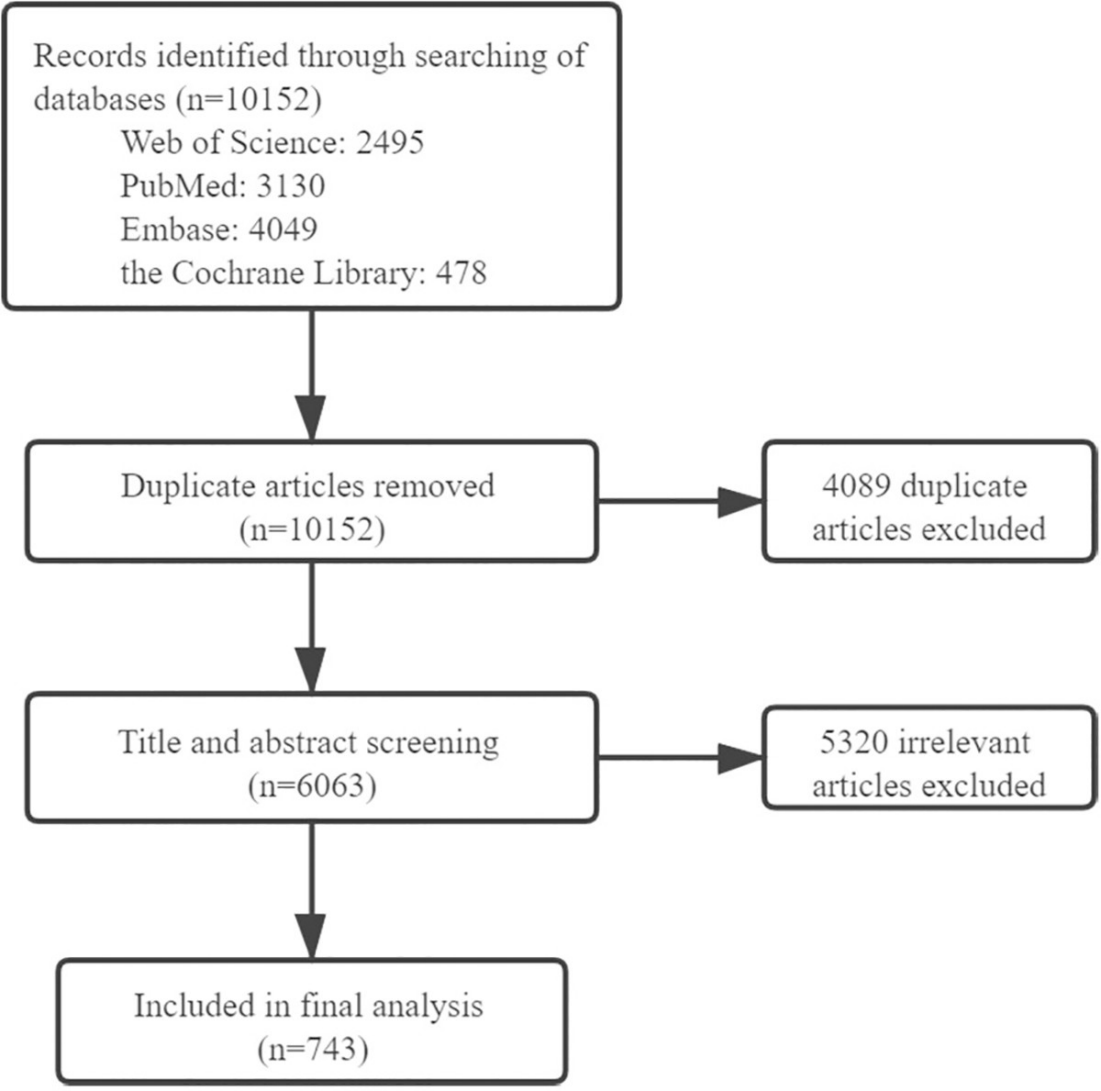
Flow diagram of included publications.

**Fig 2:**
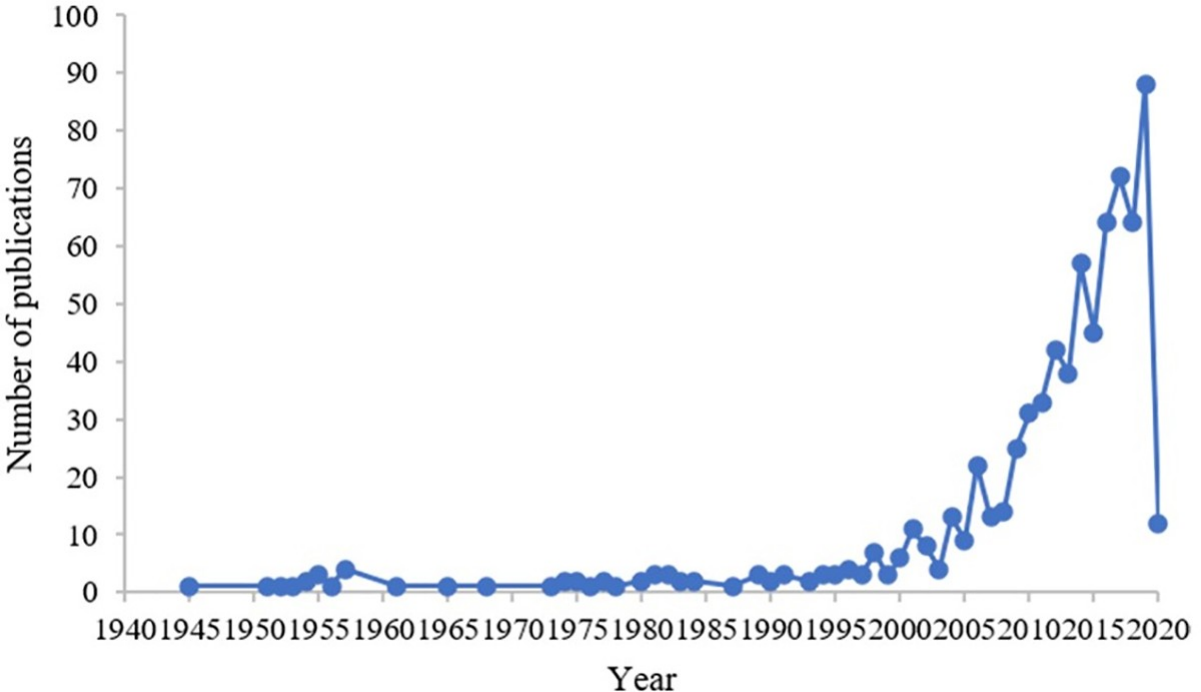
Annual number of publications per year.

A total of 10,152 publications were identified after an initial search and review. After excluding duplicates and irrelevant publications, 743 publications were retrieved for the final analysis (see Fig 1). The three main languages of these publications were English (652, 87.8%), German (20, 2.7%), and Persian (10, 1.3%). The total number of citations was 31,515 with an average of 42.4 citations per publication and an h-index of 98. The annual number of retrieved publications were shown in Fig 2. The volume of publications was small prior to 2000, after which yearly fluctuations in growth were noted. The number of articles has increased 29.3-fold since 2000.

### Countries and institutions

There were 61 countries that contributed to publications in the field of FOC. Sweden accounted for the majority (129, 16.7%), far higher than Iran which was second with 53, and the USA ranked third with 52 publications. The country with the highest number of citations per publication was Finland with 117.0 (see Table 1). The most active institutions (top 10) cited were universities or affiliated hospitals and more than half of it were in Sweden (see Table 2).

**Table 1:** The top 10 countries producing FOC-focused articles (N = 743).

Country	Number of publications	%	Number of Citations	Citations per publication
Sweden	129	17.4	10,819	83.9
Iran	53	7.1	576	10.9
USA	52	7.0	1,808	34.8
Turkey	48	6.5	1,050	21.9
UK	43	5.8	2,637	61.3
Australia	40	5.4	2,340	58.5
Norway	39	5.3	2,372	60.8
Brazil	30	4.0	407	13.6
Canada	27	3.6	1,276	47.3
Finland	23	3.1	2,692	117.0
Total	484	65.1	-	-

**Table 2:** The top 10 institutions producing FOC-focused articles (N = 743).

Institutions	Number of publications	%	Country
Karolinska Inst /Hosp	24	3.2	Sweden
Uppsala Univ/Hosp	24	3.2	Sweden
Linkoping Univ/Hosp	23	3.1	Sweden
Griffith Univ	17	2.3	Australia
British Columbia Univ	16	2.2	Canada
Helsinki Univ/Hosp	15	2.0	Finland
Mid Sweden Univ	14	1.9	Sweden
Akershus Univ/Hosp	8	1.1	Norway
Gothenburg Univ	7	0.9	Sweden
Umea Univ	7	0.9	Sweden
Total	155	20.9	-

Hosp = Hospital; Inst = Institution; Univ = University.

### Authors

Of the 2,064 authors that were found upon analyzing, the most prolific author was found to be Hildingsson, with 35 (4.7%) publications, followed by Ryding (26, 3.5%) and Fenwick (25, 3.4%) (see Table 3). In the 743 publications, rate of occurrence of contribution of co-authors was 88.4%, and the collaboration index (average number of authors per article) was 4.0. The author with the highest citations per publication was Saisto, with 132.8 citations.

**Table 3:** The top ten authors of FOC-related articles (N = 743).

Author	Number of publications	%	Number of Citations	Citations per publication
Hildingsson, I.	35	4.7	2,316	66.2
Ryding, E.L.	26	3.5	2,838	109.2
Fenwick, J.	25	3.4	1,932	77.3
Wijma, K.	24	3.2	1,799	75.0
Rubertsson, C.	22	3.0	1,180	53.6
Karlstrom, A.	21	2.8	954	45.4
Gamble, J.	20	2.7	1,425	71.3
Toohill, J.	18	2.4	683	37.9
Saisto, T.	17	2.3	2,258	132.8
Creedy, D. K.	16	2.2	1,001	62.6
Total	224	30.1	-	-

### Journals

As for these journals, *Midwifery*, *BMC Pregnancy and Childbirth*, *Acta Obstetricia et Gynecologica Scandinavica* were the most prolific journals. Almost 40% of the 743 articles were published in the top 10 most productive journals, and most of these journals specifically focused on obstetrics and gynecology (see Table 4).

**Table 4:** The top 10 journals having FOC-related publications (N = 743).

Journals	Number of publications	%	Impact factor
*Midwifery*	57	7.8	2.048
*BMC Pregnancy and Childbirth*	41	5.5	2.413
*Acta Obstetricia Et Gynecologica Scandinavica*	37	5.0	2.741
*Journal of Psychosomatic Obstetrics & Gynecology*	31	4.2	2.327
*Birth-Issues in Perinatal Care*	27	3.6	2.129
*Sexual & Reproductive Healthcare*	27	3.6	1.125
*Women and Birth*	27	3.6	2.079
*BJOG-an International Journal of Obstetrics and Gynaecology*	16	2.2	5.193
*Journal of Reproductive and Infant Psychology*	15	2.0	0.863
*Archives of Women’s Mental Health*	11	1.5	2.348
Total	289	38.9	-

### Word frequency

**Fig 3:**
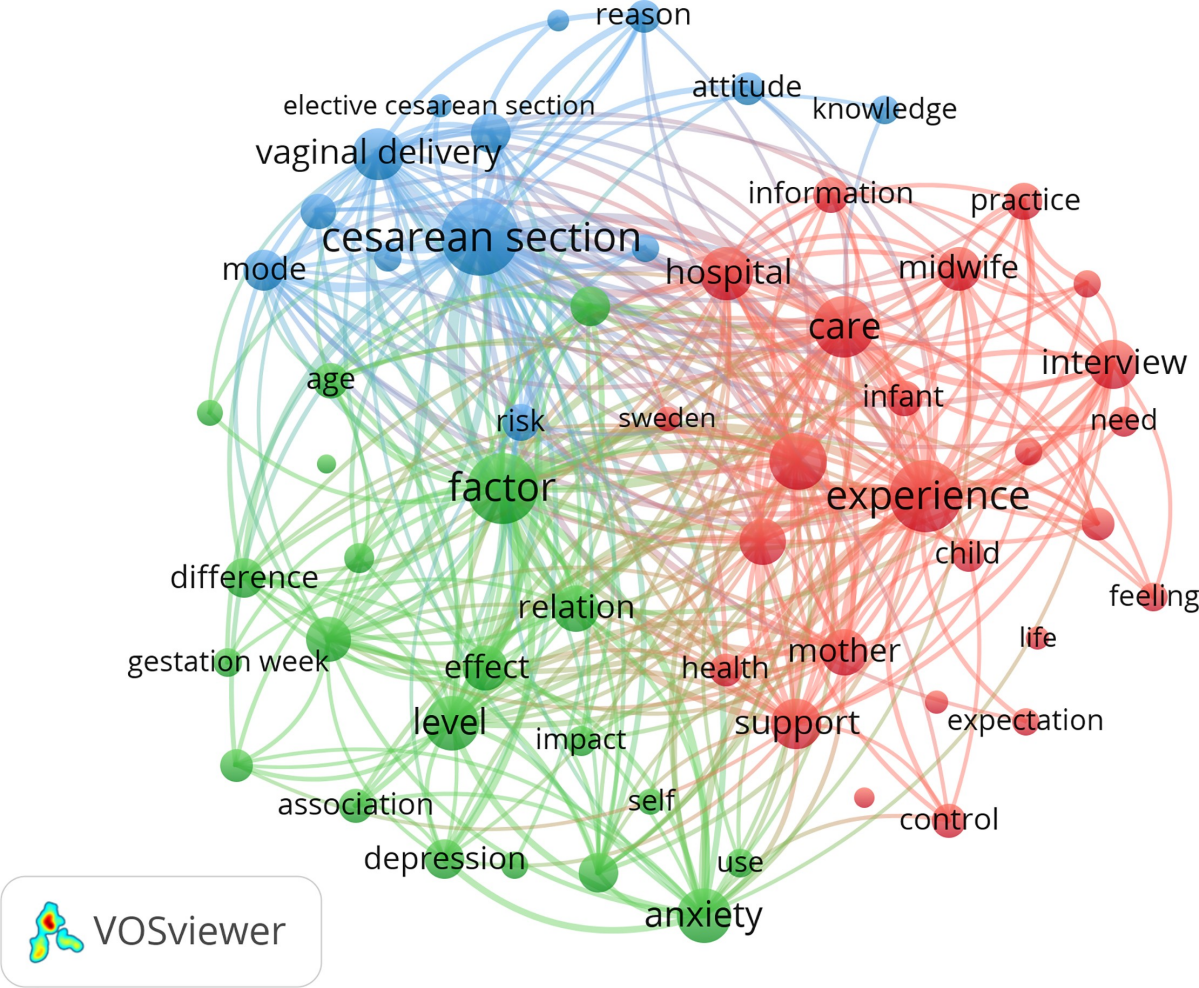
Visualization map of co-occurring words/terms in titles and abstracts having a minimum 50 occurrences of each item.

The top 20 words/terms that appear most frequently in the title/abstract of the FOC-related articles are displayed in Table 5. The words/terms occurring more than 50 times were included in the co-occurrence analysis. “Cesarean section,” “experience,” and “factor” were the three most prominent circles in the visualization map and occupied the most notable positions (see Fig 3).

**Table 5:** The top 20 words/terms appearing in the titles and abstracts (N = 743).

Word	Occurrences	%
cesarean section	309	41.6
experience	278	37.4
factor	276	37.1
care	228	30.7
pain	204	27.5
anxiety	191	25.7
level	190	25.6
hospital	185	24.9
vaginal delivery	179	24.1
support	173	23.3
interview	165	22.2
relation	157	21.1
mother	155	20.9
birth experience	153	20.6
effect	147	19.8
primiparous woman	146	19.7
midwife	144	19.4
mode	133	17.9
intervention	128	17.2
education	125	16.8

### Popularly cited articles

The top 10 cited articles are presented in Table 6. The most commonly cited article is, “Point prevalence of psychiatric disorders during the second trimester of pregnancy: A population-based study”, which has been cited 525 times [27].

**Table 6:** The top 10 cited articles in FOC-related publications (N = 743).

Author (year)	Title	Journal	Number of Citations
Andersson et al. (2003)	Point prevalence of psychiatric disorders during the second trimester of pregnancy: A population-based study	American Journal of Obstetrics and Gynecology	525
Wijma et al. (1998)	Psychometric aspects of the W-DEQ: A new questionnaire for the measurement of fear of childbirth	Journal of Psychosomatic Obstetrics and Gynecology	493
Huizink et al. (2004)	Is pregnancy anxiety a distinctive syndrome?	Early Human Development	468
Waldenstrom et al. (2006)	Antenatal fear of childbirth and its association with subsequent caesarean section and experience of childbirth	BJOG-an International Journal of Obstetrics and Gynaecology	446
Andersson et al. (2004)	Implications of antenatal depression and anxiety for obstetric outcome	Obstetrics and Gynecology	379
Hildingsson et al. (2002)	Few women wish to be delivered by caesarean section	BJOG-an International Journal of Obstetrics and Gynaecology	367
Nieminen et al. (2009)	Women's fear of childbirth and preference for cesarean section—a cross-sectional study at various stages of pregnancy in Sweden	Acta Obstetricia Et Gynecologica Scandinavica	345
Hofberg et al. (2000)	Tokophobia: An unreasoning dread of childbirth—a series of 26 cases	British Journal of Psychiatry	327
Rouhe et al. (2009)	Fear of childbirth according to parity, gestational age, and obstetric history	BJOG-an International Journal of Obstetrics and Gynaecology	323
Ryding (1993)	Investigation of 33 women who demanded a cesarean-section for personal reasons	Acta Obstetricia Et Gynecologica Scandinavica	308

## Discussion

Since the biopsychosocial model was proposed by George H. Engel, there has been a growing concern about the social, psychological and behavioral dimensions of illnesses [28]. Physical, psychological and social changes during pregnancy may lead to mental health issues, which are often not diagnosed and treated effectively [29]. The FOC is a physical and emotional state of intense anxiety, and it impairs the well-being and health of women, resulting in short-term and long-term harmful effects on mothers, infants and families [1, 30]. However, when the authors searched other maternal mental health issues such as postpartum depression in the PubMed database, the number of articles on the topic was far greater than that of FOC (by nearly eight times). To better understand the reasons for this, the current study was conducted to provide a comprehensive overview of FOC-related studies and assess the worldwide research activity on this topic.

A total of 743 publications were retrieved and analyzed in the current study. The number of FOC-related publications fluctuated at a low level prior to the 21st century, however, there has been a steady increase since 2000. A landmark article published by Wijma in 1998, introduced a psychological instrument to evaluate FOC, called the Wijma Delivery Expectancy/Experience Questionnaire (W-DEQ) and it has been recognized as the most popular tool, that has been widely used and translated into different versions [31–34]. However, when compared with other topics in the field of obstetrics and gynecology (47,811 articles on preterm birth, 6,309 on ectopic pregnancy, and 2,225 on epilepsy during pregnancy) [35–37], articles on FOC remain very low in number. In addition, there was an article with a maximum of 525 citations in this field, while there were 4,877 citations of the most commonly cited article on gestational diabetes, a popular topic in obstetrics and gynecology [38]. This indicates that the topic of FOC has not reached a mature stage compared to other similar research topics, and it deserves more attention from scholars. Among the 743 publications, the collaboration index of FOC (Index = 4.0) was smaller than areas in the field of obstetrics (Index = 5.9) [39]. Given the complexity and cost of scientific research, building a research team that works effectively is crucial [40, 41]. Fortunately, research cooperation between authors has grown over time [42]. Therefore, it is expected that this may facilitate research on FOC. Hildingsson, Ryding, and Fenwick and their colleagues were the most prolific scholars in FOC and played a pivotal role in the development and expansion of research based on the subject.

In regard to countries and institutions focusing on FOC, Sweden was the most productive country in the world. There were six institutions in the country ranking in the top 10 positions in number of publications. The topic of FOC has attracted considerable attention in Sweden from as early as 1980, with the introduction of midwife-led counseling specifically for women with FOC [43]. Furthermore, the Swedish Society of Obstetrics and Gynecology and the Swedish National Board of Health and Welfare published reports about suggestions for screening and treatment of FOC [43]. In fact, given the high incidence and serious adverse consequences of FOC, every country should lay emphasis on this health care issue, especially in areas like Asia with high birth rates. However, in the Asian continent, Iran and Turkey are the only densely populated countries that have made a greater contribution to this topic. Considering the number of citations, it is worth noting that the average number of citations in Iran, Turkey and Brazil was much lower than that in other top 10 countries. This might be due to the low impact factor of medical research journals in low-resource countries, where only 2% were in the science citation index statistics [44].

According to the volume of published articles, *Midwifery* is the most popular journal in FOC. Most of the top 10 journals are in the obstetrics and reproductive field, except for *Archives of Women’s Mental Health*. These journals provide scholars with the latest information, trends in their respective areas, and a forum for discussion while providing a suitable platform for publishing.

The most frequently occurring words/items were those involving factors related to FOC (e.g. experience, pain, support), and types of childbirth (e.g. cesarean section, vaginal delivery, mode). Compared to experimental studies, cross-sectional and qualitative studies were significantly dominant in the top 10 cited articles. Findings of bibliometric analyses appear to share information on the present situation, including causes and effects of FOC. Articles with higher number of citations overwhelmingly describe the strong link between cesarean section (CS) and FOC. Primary FOC and traumatic childbirth experiences are associated with women’s requests for CS [45, 46]. This may be due to the fact that many women who opt for a CS believe that the process will delegate unmanageable responsibilities to the medical staff and protect them from labor pain, alleviating their FOC [47]. Currently, the rate of CS keeps rising in most countries and regions of the world [48], and advocating vaginal birth as well as reducing elective CS has a global consensus [49]. Consequently, the topic of FOC deserves added emphasis.

The volume of publications on FOC has increased by nearly 30 times in the past two decades. This spike in interest in this topic indicates a greater social awareness in maternal mental health. However, through word frequency and highly cited article analysis, it was found that the content on alleviating FOC is relatively rare. Therefore, further research is needed to explore the effectiveness of existing treatment strategies, to propose the most appropriate forms of intervention, and to develop feasible and effective health care interventions in maternal health care set-ups.

This bibliometric study is the first comprehensive study of its kind. It will help future researchers to determine their priorities for their research on FOC, which can be based on this bibliometric analysis. However, there are some limitations of this study that needs to be acknowledged. Since there is no perfect search query, some relevant articles may be omitted which are inherent limitations in bibliometric analyses [19]. There were missing values in the data exported from the databases. Although the authors searched and supplemented some data in the preprocessing phase, there were still data gaps. Nevertheless, it will not have a counterfactual impact overall since such cases are rare. Organizations may have multiple names or spellings, that can result in an inaccurate analysis of productivity. Despite these limitations, the authors have tried to minimize errors by manually cleaning data as well as merging items with similar meanings.

## Conclusions

This bibliometric analysis provides an overview of FOC-related studies and identified some noteworthy issues. The topic of FOC attracts increasing attention over the world leading to a considerable increase in the number of articles published over the last two decades. This study has demonstrated that Sweden plays a leading role in FOC. The most popular topics are the affecting factors and the consequences of FOC. In the future, multi-author cooperation should be emphasized on promoting the development and progress of such research. Researchers especially in countries with high birth rates need to propose research projects on this important public health issue.

## Supporting information

S1 Appendix (DOC)

S1 File (ZIP)

S1 Data (PDF)
