# Supplementary material for: Worldwide research on fear of childbirth: A bibliometric analysis
Source: PLoS One. 2020 Jul 29;15(7):e0236567. doi: 10.1371/journal.pone.0236567 (PMC7390386; doi:10.1371/journal.pone.0236567)
Supplement: S1 Appendix — (DOC) [file pone.0236567.s001.doc]

Web of Science: (article only)

TI=((("childbirth" OR "birth" OR "delivery" OR "labor" OR "labour") AND ("fear")) OR "tokophobia" OR "tocophobia") OR AB=((("childbirth" OR "birth" OR "delivery" OR "labor" OR "labour") AND ("fear")) OR "tokophobia" OR "tocophobia")

Search date: February 10, 2020.

Result: 2495

PubMed:

((("childbirth"[Title/Abstract] OR "birth"[Title/Abstract] OR "delivery"[Title/Abstract] OR "labor"[Title/Abstract] OR "labour"[Title/Abstract])) AND "fear"[Title/Abstract]) OR ("tokophobia"[Title/Abstract] OR "tocophobia"[Title/Abstract])

Search date: February 10, 2020.

Result: 3130

Embase:

('childbirth':ab,ti OR 'birth':ab,ti OR 'delivery':ab,ti OR 'labor':ab,ti OR 'labour':ab,ti) AND 'fear':ab,ti OR 'tokophobia':ab,ti OR 'tocophobia':ab,ti

Search date: February 10, 2020.

Result: 4049

The Cochrane library: (trials only)

#1= (("childbirth" OR "birth" OR "delivery" OR "labor" OR "labour") AND "fear" OR "tokophobia" OR "tocophobia"):ab

#2= (("childbirth" OR "birth" OR "delivery" OR "labor" OR "labour") AND "fear" OR "tokophobia" OR "tocophobia"):ti

#3= #1 or #2

Search date: February 10, 2020.

Result: 478
