# Supplementary material for: Worldwide research on fear of childbirth: A bibliometric analysis
Source: PLoS One. 2020 Jul 29;15(7):e0236567. doi: 10.1371/journal.pone.0236567 (PMC7390386; doi:10.1371/journal.pone.0236567)
Supplement: S1 Data — (PDF) [file pone.0236567.s003.pdf]

CERTIFICATE OF ENGLISH EDITING

This document certifies that the paper listed below has been edited to ensure that the language is clear and free of errors. The logical presentation of ideas and the structure of the paper were also checked during the editing process. The edit was performed by professional editors at Editage, a division of Cactus Communications. The intent of the author's message was not altered in any way during the editing process. The quality of the edit has been guaranteed, with the assumption that our suggested changes have been accepted and have not been further altered without the knowledge of our editors.

Title Of The Paper

A Worldwide research on fear of childbirth in women: A bibliometric analysis.

Authors

Lijing Dai, Na Zhang, Liu Rong, Yan-Qiong Ouyang

Job Code

PCHWH_1

Signature

Vikas Narang,
Chief Operating Officer,
Editage

Date of Issue
June 16, 2020

Editage, a brand of Cactus Communications, offers professional English language editing and publication support services to authors engaged in over 500 areas of research. Through its community of experienced editors, which includes doctors, engineers, published scientists, and researchers with peer review experience, Editage has successfully helped authors get published in internationally reputed journals. Authors who work with Editage are guaranteed excellent language quality and timely delivery.

Contact Editage

Worldwide	Japan	Korea	China	Brazil
request@editage.com	submissions@editage.com	submit-korea@editage.com	fabiao@editage.cn	contato@editage.com
+1 877-334-8243	+81 03-6868-3348	1544-9241	400-005-6055	0800-892-20-97
www.editage.com	www.editage.jp	www.editage.co.kr	www.editage.cn	www.editage.com.br
